# Biomimetic Robust Starch Composite Films with Super-Hydrophobicity and Vivid Structural Colors

**DOI:** 10.3390/ijms23105607

**Published:** 2022-05-17

**Authors:** Yateng Wang, Jianru Fan, Hao Zhao, Xiaoming Song, Zhe Ji, Congxia Xie, Fushan Chen, Yao Meng

**Affiliations:** 1 College of Chemistry and Molecular Engineering, Eco-Chemical Engineering Cooperative Innovation Center of Shandong, Qingdao University of Science & Technology, Qingdao 266042, China; yateng_wang14@163.com (Y.W.); fjianru2022@163.com (J.F.); zh15140289180@163.com (H.Z.); xiecongxia@126.com (C.X.); chenfushan@qust.edu.cn (F.C.); 2 College of Marine Science and Biological Engineering, Qingdao University of Science & Technology, Qingdao 266042, China; xiaomingsong4007@163.com (X.S.); jizhe@qust.edu.cn (Z.J.); 3 Key Laboratory of Pulp and Paper Science & Technology of Ministry of Education, Qilu University of Technology (Shandong Academy of Sciences), Jinan 250353, China; 4 State Key Laboratory of Fine Chemicals, Dalian University of Technology, Dalian 116024, China

**Keywords:** biobased materials, starch composite films, cellulose fiber, high mechanical stability, structural color, super-hydrophobicity

## Abstract

The starch composite films (SCFs) will be one of the best alternative packaging materials to petroleum based plastic films, which mitigates white pollution and energy consumption. However, weak mechanical stability, water resistance, and dyeability has hindered the application of SCFs. Herein, a bioinspired robust SCFs with super-hydrophobicity and excellent structural colors were prepared by fiber-reinforcement and assembling SiO_2_/Polydimethylsiloxane (PDMS) amorphous arrays on the surface of SCFs. The properties of the designed SCFs were investigated by various methods including scanning electron microscope (SEM), Fourier transform infrared spectroscopy (FT-IR), X-ray diffraction (XRD), thermo-gravimetric analysis (TGA), a tensile test, contact angle (CA) test, and an optical test. The results showed that the obtained SCFs possessed a higher tensile strength (55.17 MPa) attributed to the formed abundant hydrogen bonds between the molecular chains of the starch, cellulose fiber, and polyvinyl alcohol. Benefiting from the nanostructure with rough surface which were modified by materials with low surface free energy, the contact angle and sliding angle of the film reached up to 154° and 2°, respectively. The colors which were produced by the constructive interference of the coherent scattered light could cover all of the visible regions by tuning the diameters of the SiO_2_ nanoparticles. The strategy in the present study not only reinforces the mechanical strength and water resistance of SCFs but also provides an environmentally friendly way to color the them, which shows unprecedented application potential in packaging materials of the starch composite films.

## 1. Introduction

White pollution is one of the most serious ecological problems, especially in the packaging field since petroleum based plastic films is highly recalcitrant to biodegradation and can contaminate the soil, rivers and seas for hundreds of years [1]. With the increasing awareness of environmental protection, “plastic limit” and biodegradable materials have become the research hotspots. Starch and cellulose are the most abundant natural polymers derived from renewable raw materials, and can be biodegraded by many kinds of microorganism [2]. So the starch composite films (SCFs) will be one of the best alternative to plastic films in packaging materials due to the high production, outstanding film-forming properties, and low cost [3]. In the packaging field, researchers have conducted lots of works to promote the application of SCFs and endow SCFs with more functions [4], such as super-hydrophobicity [5,6], high gas-barrier properties [7], anti-bacteria [8,9] and low water vapor permeability [10]. In addition, plenty of efforts have been made to employ SCFs in many other fields, including electron device, actuators [11], sensors [12], supercapacitors [13]. Though SCFs exhibited excellent performance in the fields listed above, however, the disadvantages in mechanical strength, water resistance, and dyeability hindered the practical application of SCFs as an emerging packaging material.

The mechanical stability of SCFs’ is affected by amylose proportion, starch crystallinity, and the molecular weight of the starch chains. Starch films are fragile in an anhydrous state since branched chains in starch particles tends to crystallize and lead to weak structural stability [14]. Some researchers prepared robust SCFs by using high amylose maize starch [15] and studied the effects of cationization degree on films’ properties. Lots of methods for SCFs reinforcement have been studied, such as doping different plasticizer [3], starch nanocrystals [16], inorganic filler [10], and nanocellulose [17,18]. However, these studies mostly encountered the problems including high-cost as well as inferior improvement on water resistance.

Compared to plastic films, the high-water absorption of starch films, ascribed to the abundant hydroxyl groups in polysaccharide chains, is another conundrum that puzzles researchers. Water molecules easily infiltrate into starch films and form new intermolecular hydrogen bond with starch molecular chains, which leads to swelling of pure starch films. Hence, how to enhance the hydrophobicity of SCFs has received considerable attention [5]. The hydrophobic treatment could be broadly classified into two categories: doping with some functional materials and surface modification [19]. Some emerging biodegradable polymers, such as polylactic acid (PLA), polybutylene terephthalate (PBAT) [20], polycaprolactone (PCL) and so on, were blended with starch to prepare more applicable materials, which dramatically enhanced the hydrophobicity of the SCFs. Meanwhile, the mechanical properties [21], barrier properties of SCFs were also improved [22]. In addition, multilayer-structure SCFs showed improved hydrophobicity was fabricated by combining starch layers, polyethyleneimine (PEI) and UV curable biobased acrylated epoxidized soybean oil (AESO) layer [23]. Bioinspired of the Lotus effect, the micro/nano-roughness structure was applied on SCFs surface, and the surface was further modified by materials of low surface free energy [24]. After modification, the SCFs were endowed with super-hydrophobicity which means that static contact angle of the films exceeded 150° and the sliding angle was less than 5°.

Furthermore, some well-designed patterns containing a lot of information about the product and manufacture process should be printed on the packing materials. However, the printing process cannot be completed through conventional dyeing methods, since the starch particles show inferior dissolvability in ordinary organic solvents due to the exist of the intramolecular and intermolecular hydrogen bond of the starch chains. The dye molecules cannot be grafted onto the starch chains through existing active groups. Hence, it is imperative to develop novel methods to color the SCFs. Bioinspired by the colors of some animals or plants [25], such as butterflies [26], which are produced by selective reflection, diffraction or scattering effect between the visible light and the periodic nanostructures inside their wings or skins [27,28,29], an unique coloring technique have been proposed. Acharya’s group developed a new colored starch/chitosan composite film by assembling with cellulose nanocrystal (CNC) which possessed chiral nematic structure, and the one-dimensional photonic crystal raised from self-assembly of CNC could generate different colors by regulating the helical pitch of the CNC [30]. The produced colored films exhibited reinforced UV resistance, water vapor permeability (WVP), and physical strength. Some kinds of intelligent biodegradable films with different functions were fabricated. These composite films would display various colors as the external stimuli changes [31], such as temperature [32], moisture [33], and strain [34]. However, CNC composite film is difficult to obtain and cost prohibitive. 3D colloidal crystals are arrays made up of monodispersed colloidal particles with only short-range order (amorphous arrays) or long-range order (photonic crystal) [35]. So, we could artificially construct nanostructures with specific periodic on the SCFs’ surface for endowing the packing materials excellent optical properties. In addition, modification which endows the SCFs with super hydrophobicity and colors has not been proposed in existing studies.

**Figure 1:**
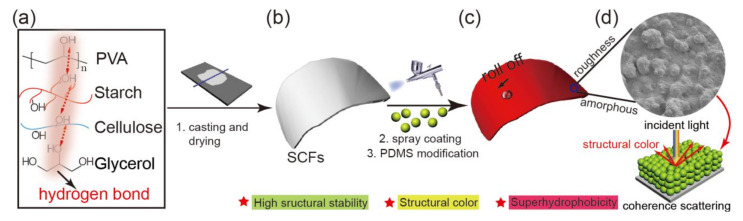
Schematic illustration showing the SPF films’ preparing process and the coloring mechanism. (**a**) Schematic diagram of intermolecular hydrogen bonding among starch, PVA and fiber in SCFs; (**b**) the SCFs prepared casting and drying; (**c**) the colored SCFs prepared by spraying SiO_2_ NPs/Carbon Black on the surface and PDMS modification; (**d**) the micro-morphology of the colored SCFs and the schematic diagram of structural color.

In this study, a kind of biomimetic robust SCFs with super-hydrophobicity and excellent structural stability was prepared successfully. The potato starch (PS) and polyvinyl alcohol (PVA) were chosen as base material. Cellulose fibers (CFs) were chosen as cost saving reinforcements to build skeleton inner the SCFs (Figure 1a,b). A vast amount of hydroxyl in starch, cellulose fibers, and PVA molecules facilitated the formation of intra- and intermolecular hydrogen bonds, favoring the combination among starch, cellulose fibers and PVA. So, the structural stability (tensile strength and elongation at break) of the Starch/PVA/fiber composite film (SPF film, prepared by casting method) had been improved significantly. Bioinspired by the Lotus effect and the structural colors in natural world, bifunctional layer with nano-roughness and colloidal amorphous arrays was constructed by spraying the silicon dioxide (SiO_2_) monodispersed nanoparticles on the surface of SCFs (Figure 1c). The SPF film reached super-hydrophobic state assisted by coating a layer with low surface energy material polydimethylsiloxane (PDMS). At the same time, the SPF film was colored by SiO_2_ colloidal crystals with different diameters and the color was produced by the interaction between the coherent scattered light and the amorphous arrays (Figure 1d). The work would offer a new pathway for the SCFs’ practical applications.

## 2. Results and Discussion

### 2.1. Morphology of the SPF Films

**Figure 2:**
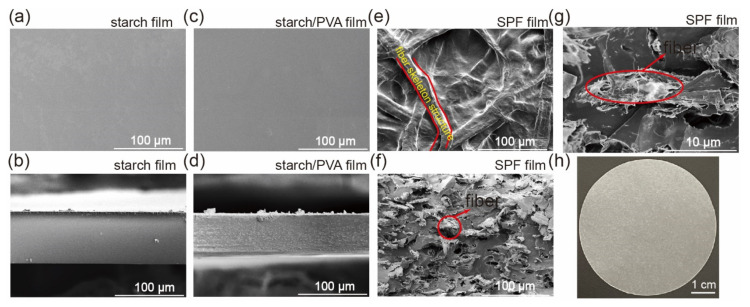
SEM images of SPF films. (**a**) Upper surface and (**b**) cross-section of starch film (S_100_P_0_F_0_). (**c**) Upper surface and (**d**) cross-section of starch-PVA composite film (S_80_P_20_F_0_). (**e**) Upper surface and (**f**,**g**) cross-section of starch-PVA-fiber composite film (S_60_P_15_F_25_). (**h**) Photos of starch-PVA-fiber composite film (S_60_P_15_F_25_).

Figure 2 shows the SEM images of SPF film and other films. There are no pores and flaws on the surface of the pure starch film and starch-PVA composite films (SEM images in Figure 2a,c), and the cross-sectional structures of both films were neat and smooth (Figure 2b,d) which demonstrated the excellent compatibility between starch and PVA. In the case of starch-PVA-fiber composite film, fibers are evenly distributed in the starch-PVA matrix and leading to a rough upper surface (Figure 2e). No obvious defections were observed between fibers and starch/PVA since abundant hydroxyl in cellulose could provide vast hydrogen bonding to build connection among fiber, starch and PVA. Similar to former researches, added fibers overlapped with each other during film formation and constructed a three-dimensional (3D) network which symmetrically filled the starch-PVA matrix [36,37]. The protruding fibers observed on the facture surface (Figure 2f,g) partially reveal the 3D network formed inside the matrix. The macroscopic morphology of the SPF film is uniform and semitransparent (Figure 2h). Owing to the intermolecular connection mentioned above, the fibers network could not only restrict molecular movement of starch and PVA under the influence of moisture or heat, but also improve the stress conduction, resulting in better performance in terms of mechanical stability and tolerance to moisture and heat.

### 2.2. FT-IR and XRD

**Figure 3:**
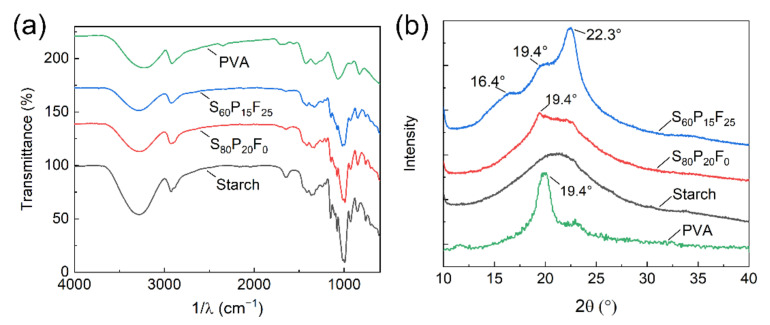
(**a**) FT-IR and (**b**) XRD pattern of different SPF films.

To investigate the chemical structure and crystalline texture of the composite films, FT-IR and XRD analysis were conducted. In the present study, the SPF films with different amount of PVA and fiber share similar FT-IR pattern (Figure 3a, Figures S1 and S2). The broad band at the wavelength of 3600 cm^−1^–3000 cm^−1^ is characteristic peak of hydroxyl stretching vibration. The peaks around 2927 cm^−1^, 1420 cm^−1^, and 1010 cm^−1^ are assigned to C-H stretching, C-H bending and C-O bending vibration, respectively [38]. The peak near 1645 cm^−1^ is related to O-H bending of water, and it diminished after the addition of PVA and fiber [39]. There is no new absorption peak detected in these composite films which indicated the presence of a hydrogen bond and Van der Waal’s force rather than a new type of chemical bond play the main role about the connection among starch, PVA, and fibers.

There are no obvious diffraction peaks on the XRD curve of starch film (S_100_P_0_F_0_, Figure 3b), which means the S_100_P_0_F_0_ film contains few crystalline regions. A sharp diffraction peak at 19.4° was observed on the curve of PVA (Figure 3b), and it grew stronger in composite films as increasing amount of PVA blended (Figure S3). This is assigned to (101) crystal plain of PVA. According to previous studies, this diffraction peak of PVA results from the strong intra- and intermolecular hydrogen bonding of PVA molecule chains [40,41]. More obvious diffraction peaks could be observed due to the further addition of fiber which has more crystalline structure. The peaks at 16.4° and 22.3° on S_60_P_15_F_25_ film curve in Figure 3b are assigned to (101), (10ī), and (002) crystal faces of cellulose I crystalline structure [42]. The diffraction peak at 34°, assigned to (040) crystal face, was distinguished in the high fiber content films (S_53_P_13_F_33_ and S_57_P_14_F_29_ in Figure S4). In general, introduction of PVA and fiber raised crystallinity of composite films, which was consistent with former researches.

### 2.3. Thermal Properties

**Figure 4:**
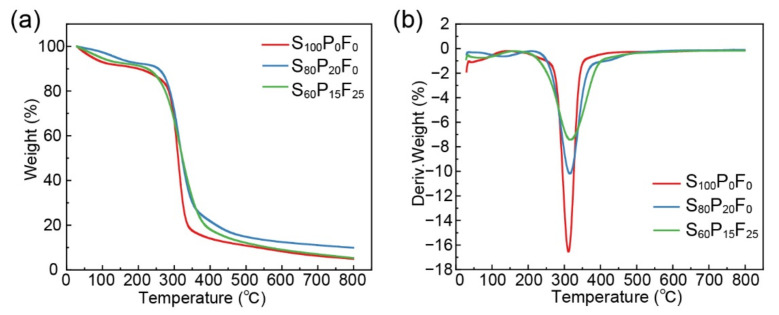
(**a**) TGA and (**b**) DTG curves of different SPF films.

The thermal properties of SPF films were evaluated by TGA and the results were displayed in Figure 4. The weight loss of starch film (S_100_P_0_F_0_) mainly occurred in two stages (Figure 4a). The first stage ended at 250 °C was assigned to the elimination of free water, glycerol, bound water, and volatile products. Then, 70% of total weight loss occurred in the second stage ranging from 250 °C to 400 °C, which was caused by degradation of starch network, including the dehydration of carbohydrate chains and the break of glycosidic bonds [43].

Mass loss curve of S_80_P_20_F_0_ film indicates different degradation process. The main weight loss stage of S_80_P_20_F_0_ film began at a lower temperature (220 °C) due to the easier dehydration of PVA. In DTG pattern of S_80_P_20_F_0_, there is a band between 400 °C and 500 °C after the main weight loss stage. As discussed in former studies, this band is ascribed to the carbonation of polyene residues [44]. More importantly, the sharp peak indicating the main weight loss in DTG curve shifts from 311 °C (S_100_P_0_F_0_) to 315 °C (S_80_P_20_F_0_), implying that PVA with higher crystallinity than starch improving the thermal stability of composite. According to the research of Senneca, the weight loss of hemicellulose started at a low temperature (180~200 °C), but the main weight loss of the cellulose occured at a high temperature due to the high crystallinity of cellulose [45]. After the addition of fiber, the start points of main weight loss kept shift toward lower temperature (200 °C), resulting from the hemicellulose in the fiber. The DTG peak increased from 315 °C to 320 °C (S_60_P_15_F_25_) due to the cellulose in the fibers. Meanwhile, the main weight loss peak became weaker and wider after addition of PVA and fibers. As mentioned in the former research, the gradual weight loss indicates better thermal stability [46].

### 2.4. Mechanical Properties

**Figure 5:**
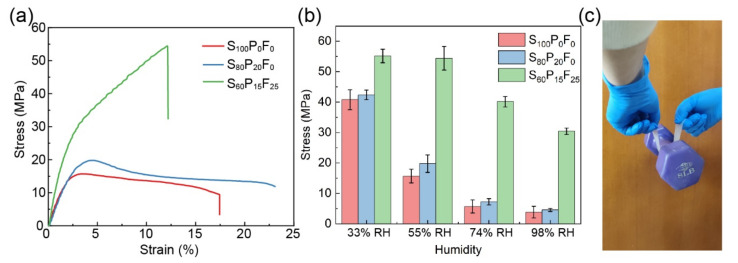
Mechanical properties of different composite films. (**a**) The strain-stress curve of composite films. (**b**) The stress of composite films under different humidity. (**c**) The digital image of the robust SPF films carrying a barbell.

The influence of PVA and fiber on mechanical properties of SPF films is revealed in Figure 5, Figures S5 and S6. The results showed that the addition of PVA simultaneously increased stress and elongation at break of the composite film, and the higher PVA content led to the higher stress and elongation (Figure 5a and Figure S5). The tensile strength was further enhanced while elongation was almost halved when introducing fiber into starch-PVA matrix. The fiction between fibers or between fiber and starch-PVA matrix afforded extra force to resist external stress [47]. However, the fiber network inside starch-PVA matrix hindered the free movement of starch/PVA molecular and might impair ductility of composite film. In addition, the fiber content plays a crucial role on mechanical properties as fiber flocculation at the high fiber consistency during film preparation weakened the stress conduction of the composite film, which explains the impaired mechanical performance as long as the fiber content surpassed 25 wt% (Figures S6 and S7) [48]. According to the former study, the fiber network restricts the movement of starch/PVA molecular and reduces the number of free hydroxyls, the invasion of water molecular can be blocked [49]. Hence, after the introduction of fiber, the SPF film preserved high mechanical strength under high humidity circumstances. As shown in Figure 5b, the maximum stress of the S_100_P_0_F_0_ film and S_80_P_20_F_0_ film reduced from over 40 MPa under 33% RH to 15.72 Mpa and 19.8 Mpa under 55% RH, and further reduced to below 10 Mpa under 74% RH and 98% RH. However, the S_60_P_15_F_25_ film whose maximum stress was 55.17 Mpa under 33% RH and 54.42 Mpa under 55% RH possessed maximum stress of 40.14 Mpa at 74% RH and 30.44 Mpa at 98% RH. Hence, the SPF composite films possess excellent adaption to a wider humidity range. An 8LB barbell could be lifted with the SPF film (2 cm wide) easily (Figure 5c).

### 2.5. Colored SPF Films and Their Optical Properties

Bioinspired by the structural colors which exist in the natural world, the amorphous arrays with only short-range order which could endow the film excellent optical properties has been constructed on the surface of SPF films. The super-hydrophobicity could be realized simultaneously by coating polydimethylsiloxane (PDMS) on the rough surface of the amorphous arrays. Monodispersed SiO_2_ nanoparticles (SiO_2_ NPs) with various diameters (210 nm, 245 nm, 276 nm, 293 nm and 322 nm) were prepared successfully and chosen as the construct unit. The five kinds of synthesized SiO_2_ NPs showed outstanding monodispersity and spherical morphology (SEM images in Figure S8). The polydispersity indexes (PDI) of the obtained SiO_2_ NPs were all less than 0.05 which demonstrated the uniformity of the SiO_2_ NPs. The zeta potentials of the SiO_2_ NPs were all bigger than −30 mV (Table S1).

**Figure 6:**
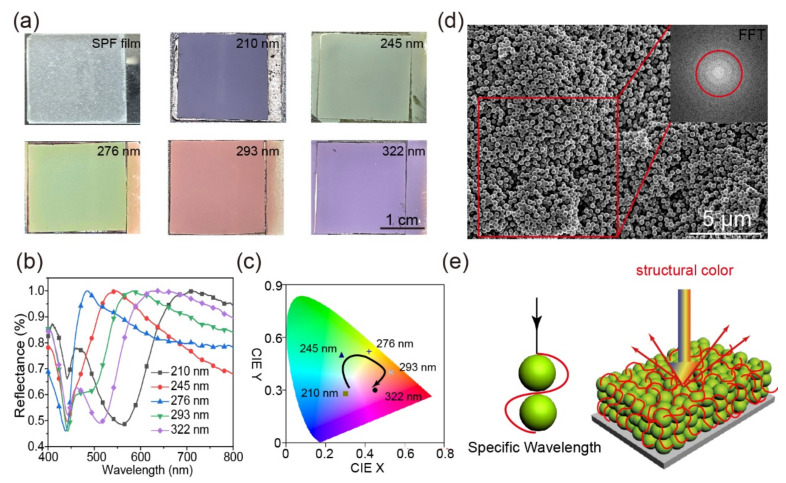
(**a**) The digital images of the colored SPF films. (**b**) The reflectance spectra and (**c**) CIE chromaticity diagram of the colored films. (**d**) The SEM images and corresponding FFT pattern of the amorphous arrays. (**e**) Schematic illustrate of the coloration mechanism.

The SPF films exhibit vivid structural colors after the SiO_2_ NPs/CB dispersion spraying process and PDMS modification. The abundant colors were uniform, bright, and highly saturated, which could cover all over the visible region (Figure 6a). The saturation of the structural colors was enhanced significantly since CB could absorb the incoherent scattered light across the entire visible region. The reflection peaks (reflection spectra in Figure 6b) sticked out at 470 nm, 540 nm, 583 nm, 628 nm, and 705 nm according to the various diameters. The spectra were transferred to more standard Commission Internationale de L’Eclairage (CIE) values (Figure 6c), and the biomimetic colors covered the full-color range which were demonstrated by the reflection and CIE spectra. The arrange characteristics of the amorphous arrays were shown in the SEM image (Figure 6d) and the corresponding fast Fourier transform (FFT) pattern revealed the arrays’ spatial information. A series of symmetrical concentric rings could be observed in the FFT pattern (red circle in Figure 6d), the circular spectra means that the arrangement of the SiO_2_ NPs is isotropy and with only short-range order. As the schematic illustrates (Figure 6e), the color was produced by the constructive interference of the coherent scattered light, and the scattering light with specific wavelength was determined by the diameter of the several adjacent SiO_2_ NPs.

### 2.6. Superhydrophobicity of SPF Films

**Figure 7:**
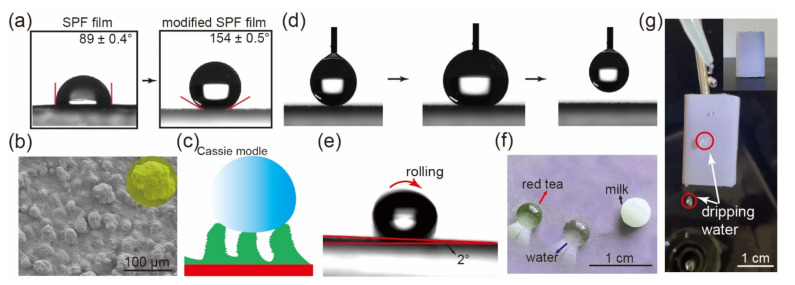
(**a**) CAs of unmodified SPF films and modified SPF films. (**b**) SEM image of the modified composite film surface. (**c**) Scheme diagram of lotus super-hydrophobicity. (**d**) The adhesion of the film. (**e**) Sliding angle of modified composite film. (**f**) Digital photo of the droplet of different liquid on the surface of the films. (**g**) The digital image of the dripping water of the SPF packing box.

SPF films with biomimetic vivid structural colors and super-hydrophobicity were fabricated by assembling SiO_2_ NPs/PDMS on the surface of the composite films. After the modification, the contact angles (CAs) promoted from 89° to 154° (Figure 7a). The surface morphology of colored SPF films with different quantity of SiO_2_/ethanol dispersion and PDMS was shown in Figures S9 and S10. The results showed that the surface roughness, as well as the number and size of humps on the surface, dramatically augmented along with the increasing spraying quantity of SiO_2_/ethanol dispersion, and that 0.02 mL/cm^2^ was the best spraying quantity (CA = 154°). Appropriate coating content of PDMS was important for the super-hydrophobicity of the colored SPF films since SiO_2_ nanoparticles was sticked together and the gaps between SiO_2_ nanoparticles were fulfilled by PDMS when the dip repeated for three times, which reduced the microstructure of the humps (roughness) and CAs (Figure S10d).

The surface of the composite film modified by SiO_2_ nanoparticles and PDMS displayed plenty of hydrophobic humps which resembled the micro-structure of lotus leaf surface and endow the composite film super-hydrophobicity (Figure 7b,c). Some air was trapped in the humps of the SiO_2_/PDMS arrays. The phenomenon fits the Cassie and Baxter model (*cosθ_r_ = f_sol_cosθ_fla_* − *f_air_*) very well which describe the relationship between the CAs of a water droplet on a heterogeneous surface (*θ_h_*) and flat surface (*θ_fla_*). In the model, the *f_sol_* and *f_air_* represent the area proportion of the SiO_2_/PDMS surface and air contact with the water droplet. The water CAs of the flat (*θ_fla_*) and the rough SiO_2_/PDMS surface film (*θ_r_*) are 110° and 154°. *f_air_* is about 0.847, which means the super-hydrophobicity is mainly attributed to the air trapped in the SiO_2_/PDMS humps and cavities in the rough coating surface. Besides, the modified surface showed extremely low adhesion. A big drop of water (15 μL) was dripped onto the film and the water drop could not wet the surface. There was no residue when the water droplet was pipetted away (Figure 7d) and the sliding angle was only 2° (Figure 7e), which also demonstrates the low adhesion and the super-hydrophobicity of the SPF films. The surface was resisted to the contamination of common liquid in daily life, such as milk and tea (Figure 7f). To demonstrate the practical application of the films, a packing box (inset in Figure 7g) was prepared with the modified SPF films, and the water drops slid quickly without any residual (Figure 7g). The video of the dripping water has been included in the Supplementary Materials.

## 3. Materials and Methods

### 3.1. Materials

The bleached hardwood pulp fiber (moisture content of 7.6%, lignin content of 1.3%, hemicellulose content of 5.7%, and cellulose content of 84.9%) was kindly provided by China National Pulp and Paper Research Institution Co., Ltd. (Beijing, China). The potato starch with moisture content of 13.3%, amylose content of 19.8% and crystallinity of 25.1% was purchased from Sinopharm Chemical Reagent Co., Ltd. (Shanghai, China). Poly(vinyl alcohol) 1799 (alcoholysis degree 98~99%) was purchased from Shanghai Macklin Biochemical Co., Ltd. (Shanghai, China). Polydimethylsiloxane (PDMS) was got form Dow Corning Co., Ltd. (Midland, MI, USA). Tetraethoxysilane (TEOS), ammonium hydroxide (NH_3_·H_2_O, 25wt%), ethanol and n-hexane were also bought from Sinopharm Chemical Reagent Co., Ltd. (Beijing, China) as analytical reagents.

### 3.2. Preparation of the SPF Films

The air-dried bleached hardwood pulp fiber was first soaked in distilled water for 5 h and then defibered for 5 min in a standard pulp disintegrator (IMT-SJ02, Dongguan, China) at consistency of 1.0 wt%, which furthest reduced the fiber agglomeration. The disintegrated fiber was obtained by filtration and kept at a consistency of below 20%. Then, potato starch, PVA, disintegrated fiber, and distilled water were packed into a flask to form a 5 wt% suspension, following the addition of glycerol which served as plasticizer. The composition of the suspensions differed in different experiment groups and the composition information of the suspension was represented by a designation S_X_P_Y_F_Z_ films. For example, S_60_P_20_F_20_ refers to a suspension containing 60 wt% starch, 20 wt% PVA and 20 wt% fiber as its suspended solid. The detailed grouping and composition information were listed in Supplementary Materials (Table S2). The complete dispersion of the fiber was realized under an agitation at a speed of 400 rpm and room temperature for 10 min. Subsequently, the mixture was heated to 95 °C in a water bath accompanied with 500 rpm stirring. After 60 min, the suspension was transformed to a uniform translucence casting solution. A membrane casting equipment (BEVS 1811 Automatic Film Applicator, Guangdong, China) was utilized to cast the solution into wet film on a polystyrene board. The wet film was dried at 50 °C for 120 min in an oven. The thickness of composite films was regulated by adjusting the blade clearance of the film casting equipment and the thickness of the obtained films was 90 ± 3 μm.

### 3.3. Preparation of the SiO_2_ Monodispersed Nanoparticle

The SiO_2_ monodispersed nanoparticles were synthesized through modified *Stöber* method. 50 mL absolute ethanol, 100 mL deionized water and NH_3_·H_2_O (28 wt%) with various amounts (5–25 mL) were added as the mixed solvent into a 250 mL three necked flask which was placed in a 25 °C water bath with magnetic stirrer. After stirring for 5 min at 2000 rpm, the mix solution containing 16 mL TEOS and 64 mL absolute ethanol was added into the flask. After 2 min, the stirring speed was descended to 700 r/min. The reaction went on for 4 h. Then the crude product was centrifugally washed with deionized water and anhydrous ethanol for three times to obtain pure SiO_2_ nanospheres. The SiO_2_ product was dispersed in ethanol (7 wt%) to form uniform SiO_2_/ethanol dispersion for further use.

### 3.4. Preparation of the Colored SPF Film with Super-Hydrophobicity

The SPF films with non-iridescent structural colors were prepared through spray-coating method by using an airbrush (with 0.2 mm diameter) driven by pressurized air. SiO_2_ nanoparticles were first sprayed onto the composite film to form a rough surface. The spraying quantity of SiO_2_/ethanol dispersion ranged from 0.01 mL/cm^2^ to 0.04 mL/cm^2^. The colors of the colored SPF films were regulable by using SiO_2_ NPs with different diameters, and the dosage of SiO_2_/carbon (CB) dispersion was 0.3 mL/cm^2^. Then, the films were dipped into 2 wt% PDMS/n-hexane solution to gain the film a hydrophobic coating. The dip process which lasted 5 s each time was repeated 0–3 times to regulate the PDMS content on the surface of composite films. Finally, the films were placed in a 100 °C oven for solidifying the PDMS (30 min).

### 3.5. Characterization

Fourier transform infrared spectroscopy (FT-IR) was investigated using Thermo Scientific Nicolet iS20 under spectrometer under ATR mode. A Rigaku Ultima IV X-ray diffraction (XRD) instrument (Rigaku, Tokyo, Japan) was used to conduct XRD analysis of samples. The testing was carried out with scan rate of 2°/min and diffraction angle (2θ) range of 5° to 60°. The micro-images of composite films were observer by a Hitachi SU-8100 field emission scanning electron microscope, operating at an acceleration voltage of 5 kV. Thermal gravity analysis (TGA) was conducted on a NETZSCH (TGA500) thermal analyzer under nitrogen atmosphere. The temperature range in the test was from room temperature to 800 °C, and the heating rate was set to 10 °C/min.

Water CA measurements were conducted using Lauda LSA60 tester. For every kind of composite film, five pieces were prepared for repeated tests to guarantee the precise results. The reflectance spectra of the non-iridescent SPF films were measured by HITACHI U-4100 under diffuse reflection model.

Mechanical properties of composite films were investigated by a ZWICK Z005 universal tensile testing machine. The initial gap separation and crosshead speed were adjusted to 50 mm and 5 mm/min, respectively. All composite films, tailoring into 80 mm × 15 mm pieces, were placed in desiccators containing magnesium nitrate (55% RH) saturated solution for one week before tensile testing. When mechanical strength under different humidity circumstance was studied, the sample pieces was placed in desiccators containing magnesium chloride (33% RH), magnesium nitrate (55% RH), sodium chloride (75% RH), and copper sulfate (98% RH) saturated solution for one week before tensile testing.

## 4. Conclusions

A kind of bioinspired colored starch/PVA/fiber composite films with super-hydrophobicity and excellent structural stability has been prepared successfully. Benefitting from the stress conduction and restriction of the fiber skeleton in the starch/PVA matrix, the S_60_P_15_F_25_ film showed preferable tensile strength (a maximum stress of 55.17 MPa under 33% RH) and excellent adaption to high humidity circumstance. The outstanding optical properties and super-hydrophobicity were endowed to the SPF film by assembling a bifunctional layer on the surface of the SPF films. The vivid structural color could cover the entire visible range, which was produced by the constructive interference of the coherent scattered light. As a result of the rough surface formed by SiO_2_ NPs and modification with moderate amount of PDMS, the CAs and sliding angle of the super-hydrophobic film was 154° and 2°, respectively.

## Data Availability

Not applicable.

## Supplementary Materials

The following supporting information can be downloaded at: https://www.mdpi.com/article/10.3390/ijms23105607/s1.
