# Supplementary material for: Biomimetic Robust Starch Composite Films with Super-Hydrophobicity and Vivid Structural Colors
Source: Int J Mol Sci. 2022 May 17;23(10):5607. doi: 10.3390/ijms23105607 (PMC9145899; doi:10.3390/ijms23105607)
Supplement: Supplementary file 1 [file ijms-23-05607-s001.zip › ijms-1718971-supplementary/Supporting Documents/Supporting Information.pdf]

Biomimetic Robust Starch Composite Films with Super-hydrophobicity and Vivid Structural Colors

Yateng Wang [a,b], Jianru Fan [a], Hao Zhao [a], Xiaoming Song [a], Zhe Ji [a], Congxia Xie [a], Fushan Chen [a], Yao Meng [a, b, c*]

[a.] College of Marine Science and Biological Engineering, College of Chemistry and Molecular Engineering, Eco-chemical Engineering Cooperative Innovation Center of Shandong, Qingdao University of Science & Technology, Qingdao 266042, PR China

[b] Key Laboratory of Pulp and Paper Science & Technology of Ministry of Education, Qilu University of Technology (Shandong Academy of Sciences), Jinan, 250353

[c.] State Key Laboratory of Fine Chemicals, Dalian University of Technology, Dalian 116024, PR China

* Corresponding author.
E-mail addresses: mengyao@qust.edu.cn, Qingdao University of Science & Technology, 53# Zhengzhou Rd, Qingdao 266042, PR China.

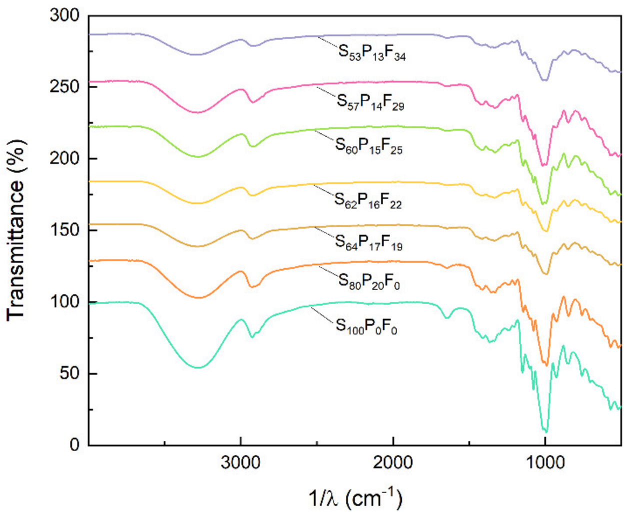

Figure S1. FT-IR spectra of starch-PVA-fiber composite films with different fiber content

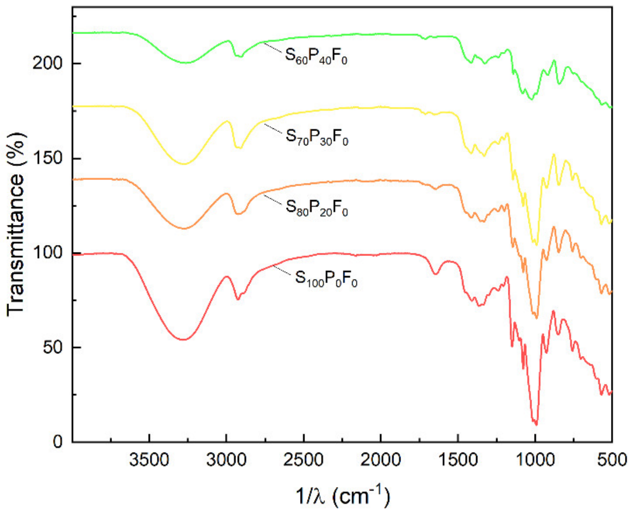

Figure S2. FT-IR spectra of starch-PVA composite films with different PVA content

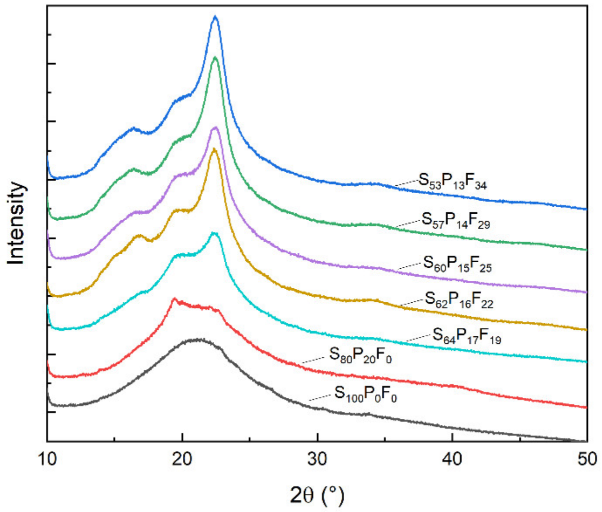

Figure S3. XRD spectra of starch-PVA-fiber composite films with different fiber content

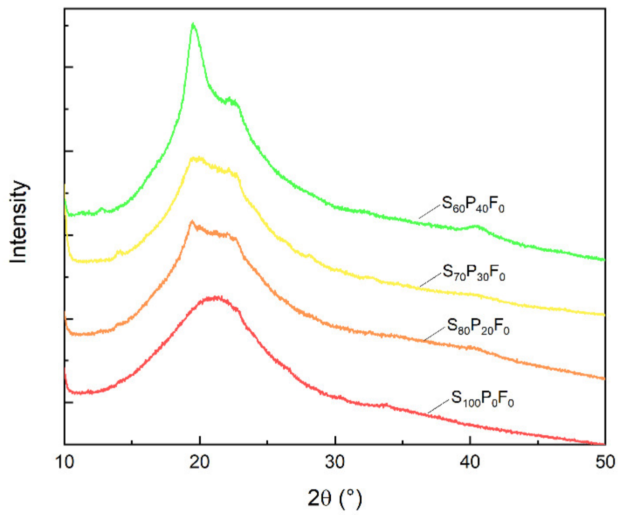

Figure S4. XRD spectra of starch-PVA composite films with different PVA content

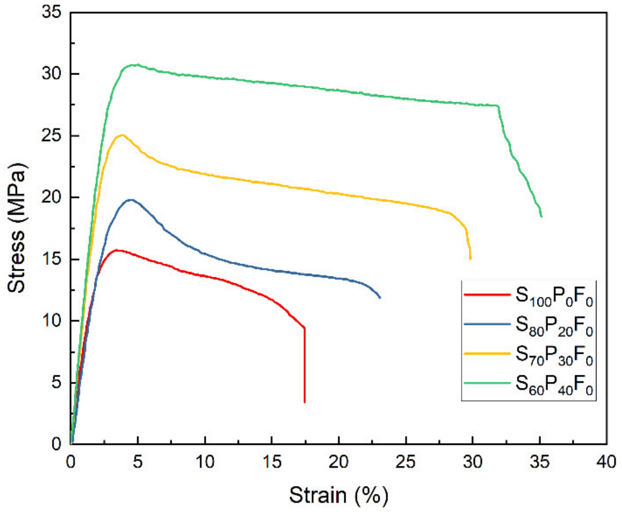

Figure S5. Stress-strain curves of starch-PVA composite films with different PVA content

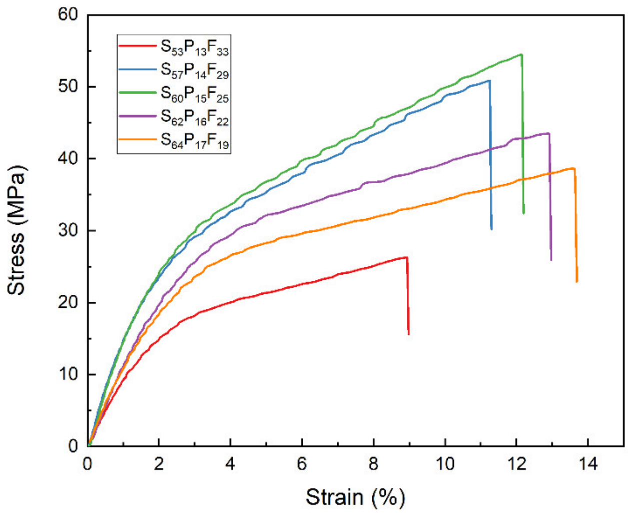

Figure S6. Stress-strain curves of starch-PVA-fiber composite films with different fiber content

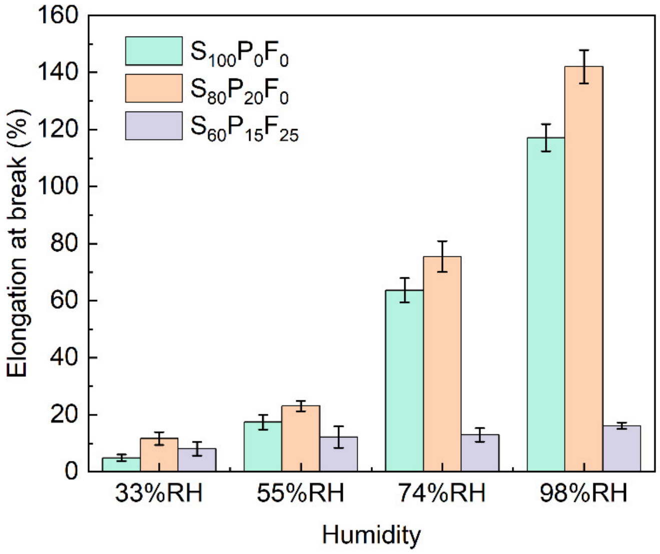

Figure S7. The elongation at break of composite films under different humidity

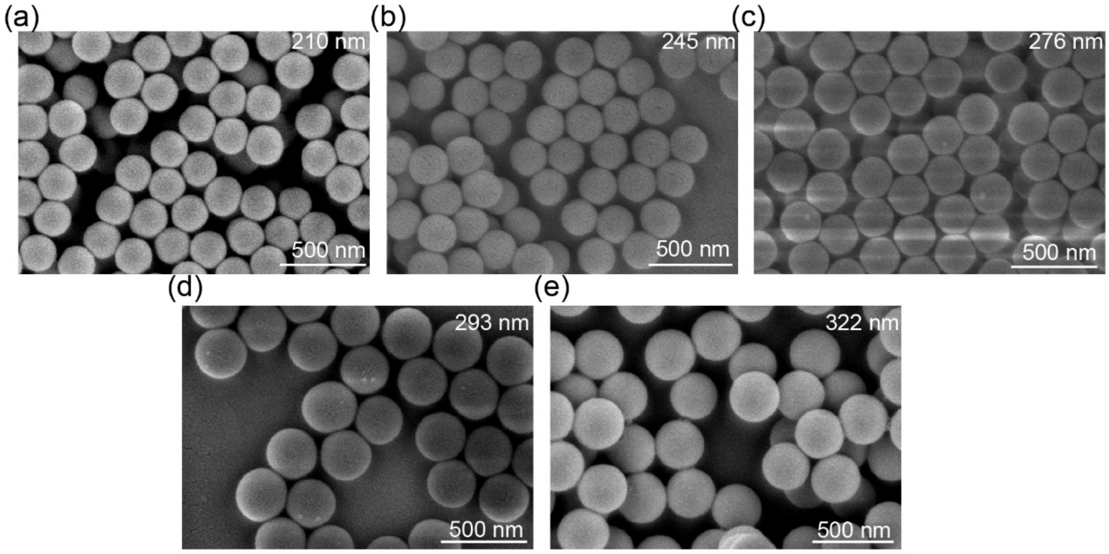

Figure S8. The monodispersed SiO_2 nanoparticles with diameters of (a) 210 nm, (b) 245 nm, (c) 276 nm, (d) 293 nm and (e) 322 nm, respectively.

Table S1. Diameters and Zeta-potential of Six Silica Nanospheres Samples

Sample	$NH_3 \cdot H_2O$ Dosage (mL)	Size (nm)	PDI	Zeta Potential (mV)
(a)	5	210	0.037	-33.8
(b)	10	245	0.026	-31.1
(c)	15	276	0.019	-35.3
(d)	20	293	0.030	-39.3
(e)	25	322	0.041	-42.6

Table S2. Coding nformation and formulation of composite films

Code	Starch (o.d.) / g	PVA (o.d.) / g	Fiber (o.d.) / g	Glycerol (o.d.)[a] / g
$S_{100}P_0F_0$	10.00	0	0	2.00
$S_{80}P_{20}F_0$	8.00	2.00	0	1.60
$S_{70}P_{30}F_0$	7.00	3.00	0	1.40
$S_{60}P_{40}F_0$	6.00	4.00	0	1.20
$S_{64}P_{17}F_{19}$	6.40	1.70	1.90	1.28
$S_{62}P_{16}F_{22}$	6.20	1.60	2.20	1.24
$S_{60}P_{15}F_{25}$	6.00	1.50	2.50	1.20
$S_{57}P_{14}F_{29}$	5.70	1.40	2.90	1.14
$S_{53}P_{13}F_{34}$	5.30	1.30	3.40	1.06
$S_{52}P_{23}F_{25}$	5.20	2.30	2.50	1.04
$S_{45}P_{30}F_{25}$	4.50	3.00	2.50	0.90

a: the addition of glycerol was based on mass (o.d.) of potato starch.

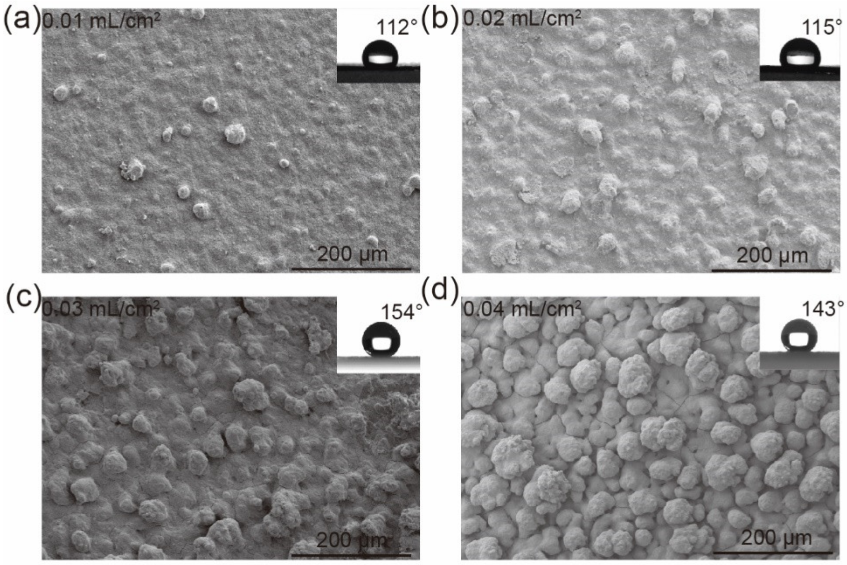

Figure S9. The surface morphology and CAs changes of the SPF films with different spraying quantity of SiO_2/ethanol dispersion.

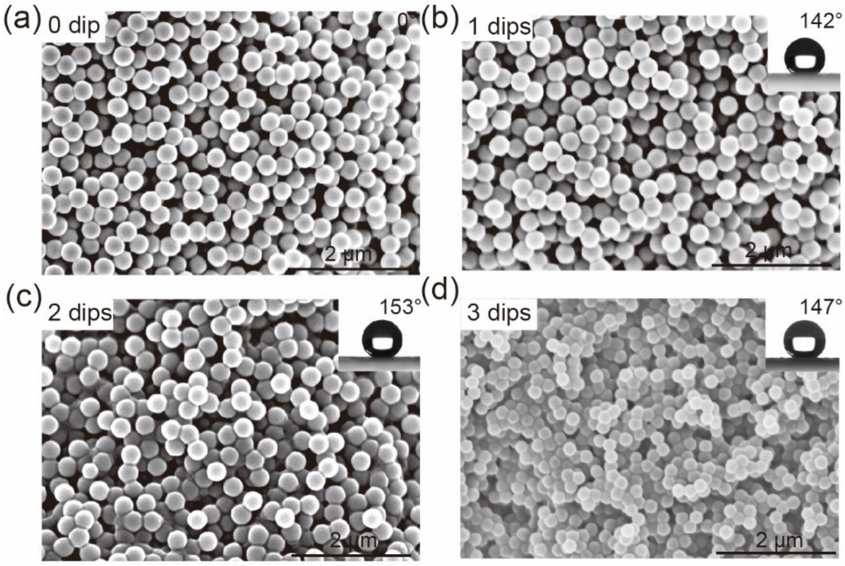

Figure S10. The surface morphology and CAs changes of the SPF films with different dipping times in PDMS/n-hexane dispersion.

The video of the dripping water was listed in Video S1.
